# “Dirty Dry Eye”– A waste volume analysis from topical therapy in keratoconjunctivitis sicca

**DOI:** 10.1007/s00417-024-06431-y

**Published:** 2024-03-23

**Authors:** Alexandra V. Schilcher, Mathias Roth, Friedrich A. Steindor, Ranya Helweh, Gerd Geerling

**Affiliations:** Department of Ophthalmology, University Hospital Duesseldorf, Duesseldorf, Germany

**Keywords:** CO_2_ footprint, Dry eye disease, Multi-dose units, Waste, Single-dose units, Sustainability

## Abstract

**Purpose:**

The healthcare system is responsible for around 5% of CO_2_ emissions globally and in Germany. So far, there are no data on the amount of waste from dry eye disease (DED) therapy in ophthalmology. The aim of this project was to evaluate the amount and type of waste from single- and multi-dose units (SDU/MDU) generated by eyedrops used to treat DED in Germany.

**Methods:**

The net waste weight (outer/inner packaging, instruction leaflet, empty container) from factory-sealed products was determined using a precision scale. Based on prescription data from PharMaAnalyst, a database of medical prescriptions from over 70 million patients in Germany, the total annual waste volume for 2016–2021 and the net weight of a 30-day treatment were calculated.

**Results:**

The total annual waste volume increased significantly (*p* < 0.0001) from 7.13 tons in 2016 to 20.64 tons in 2021. A 30-day treatment with MDUs (without/with filter) results in a significantly lower mean waste volume (paper: SDU 24.3 ± 18.7 g; MDU 4.8 ± 1.7 g/8.8 g ± 1.7 g; SDU/MDU *p* = 0.0003, with filter *p* = 0.0034; plastic: SDU 35.0 ± 4.0, MDU 6.6 ± 0.7 g/ 15.1 g ± 5.8 g, SDU/MDU *p* < 0.0001, with filter *p* < 0.0001).

**Conclusion:**

Prescription-based treatment of DED in Germany causes an increasing and substantial waste volume. The use of SDUs is considerably more resource-intensive than MDUs. Due to the large and rising number of patients suffering from DED improvements in packaging could considerably reduce the CO_2_ footprint of DED treatment.


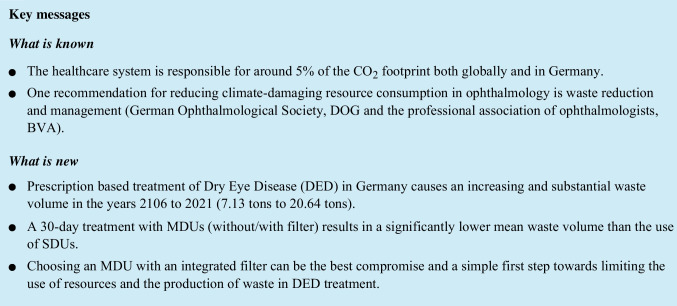


## Introduction

The healthcare system is responsible for around 5% of the CO_2_ footprint both globally and in Germany [1–4]. A rapid and substantial reduction in CO_2_ emissions over the next few years is required to meet the goals of the binding 2015 international agreement to mitigate worldwide climate change [5]. It is likely that ophthalmology is responsible for a substantial share of CO_2_ emissions due to intensive or chronic treatment of large patient numbers, e.g., in patients in need of repeated intravitreal injections for neovascular macular degeneration, cataract surgery, or chronic topical medication in glaucoma or dry eyes disease. Patient safety and quality of care provided, an ecologically sustainable, climate-neutral ophthalmology are the goals for the coming decades.

In 2023, the German Ophthalmological Society (Deutsche Ophthalmologische Gesellschaft, DOG) and the professional association of ophthalmologists (Berufsverband der Augenärzte, BVA) defined recommendations for an ecological and sustainable ophthalmology [6]. One recommendation for reducing climate-damaging resource consumption in ophthalmology is waste reduction and management [7]: wherever possible, strategies for reducing waste volume should be developed. These can be developed following the 5R model by reducing, reusing, recycling, rethinking, and researching resources and materials [8]. “Doing more with less” is the most effective concept of reducing resources and producing less waste [9].

Dry eye disease (DED) is one of the most common diagnoses in ophthalmology. The prevalence is 15 to 17% in the general population of Germany, corresponding to more than 12 million people. In Europe and the USA, the prevalence has been reported to vary between 5 and 15%, depending on age, sex, and severity of the disease [10, 11]. The prevalence is further increasing due to the increasing use of display devices [12], the aging population [11], and environmental factors such as pollution and climate change [13].

The basic therapy of DED for both the aqueous-deficient and evaporative forms consists of the regular and often long-term application of artificial tears or anti-inflammatory drugs [14, 15]. These products are regularly aliquoted in single or multidose units (SDU/MDU) of plastic, packaged in cardboard, and contain instructions on paper. Depending on disease severity and product parameters such as volume per vial or preservation method, patients are likely to produce a substantial volume of plastic and paper or cardboard waste each month.

So far, the CO_2_ footprint of topical forms of treatment in DED has never been analyzed in detail. As an indirect measure of CO_2_ footprint, our goal was to assess the waste volume of topical therapy in DED based on current prescription figures in Germany.

## Material and methods

### Prescription data

PharMaAnalyst, a freely accessible database by the scientific institute of the largest German health insurance (Allgemeine Ortskrankenkasse, AOK), was used to determine the name and number of eyedrops prescribed annually for DED. PharMaAnalyst is based on the drug index of the statutory health insurance (SHI) in Germany.

This SHI-drug index contains prescription data of more than 185,000 different drugs for over 70 million people with SHI, thus covering 83% of the 84 million people currently living in Germany/German inhabitants [16]. Data on all drug prescriptions issued by doctors to insured persons, which are billed by public pharmacies and hospital pharmacies in outpatient care at the expense of the SHI, is gathered. This currently involves an annual volume of approximately 820 million individual prescriptions. Not included in this data are non-prescription drugs, unless they are reimbursed by the SHI—e.g., for children—and other private purchases in self-medication (“over the counter,” OTC drugs) as well as private prescriptions [17]. For market analysis, the data of the 3000 drugs with the highest prescriptions, daily doses, and sales in the selected year is processed and available for individual analyses. This enables valid statements to be made about the quantities and costs of around 98% of all drug prescriptions in a given year for the more than 70 million people insured by the SHI system [17]. For the number of prescribed packs of a drug, each pack is counted. The search in the PharMaAnalyst database (October 3rd, 2023; search keywords: year 2016–2021, Artelac^®^, Corneregel^®^, Euphrasia eyedrops Weleda/Wala^®^, Hylo Gel^®^, Ikervis^®^, Vismed/-light/-multi^®^, Softacort^®^) resulted in a list of seven products (six different ingredients: hypromellose, dexpanthenol, euphrasia, haluronic acid, ciclosporin A, and hydrocortisone) used in the treatment of DED, which are available in a total of 29 different packaging sizes/application forms (18 SDU and 6 MDU without filter and 5 MDU with filter) (Table 1). MDUs without filter include preservatives (Vismed light^®^ 15 ml, Artelac Complete^®^ 10 ml, and Corneregel Fluid^®^ 10 ml). The dosage forms and package sizes (N1–N3) contained in one pack were determined with a detailed annual listing for the products, provided by PharMaAnalyst.

**Table 1:** Overview of products and ingredients analyzed. (A) 18 SDUs, (B) MDUs (6 MDUs without filter and 5 MDUs with filter). # MDU with filter. * prescription mandatory.

Active ingredient	Product	Package size	Paper weight	Plastic weight
**A**				
Hyaluronic acid	Vismed^®^ (TRB Chemedica, Staffordshire, UK)	N1 (20 SDU)	18.96 ± 0.00	23.66 ± 0.00
		N2 (60 SDU)	29.82 ± 0.00	70.90 ± 0.01
		N3 (120 SDU)	45.13 ± 0.00	136.24± 1.57
Ciclosporin A*	Ikervis^®^ (Santen SA, Genf, Switzerland)	N1 (30 SDU)	17.35 ± 0.00	41.90 ± 0.00
		N2 (90 SDU)	36.08 ± 0.00	123.86 ± 2.73
Hydrocortisone*	Softacort^®^ (Théa PHARMA SA, Schaffhausen, Switzerland)	N1 (30 SDU)	15.05 ± 0.02	40.50 ± 0.00
Euphrasia	Euphrasia^®^ (WELEDA AG, Arlesheim, Switzerland)	N1 (5 SDU)	7.43 ± 0.00	5.22 ± 0.00
		N1 (10 SDU)	8.02 ± 0.00	10.69 ± 0.00
		N1 (20 SDU)	8.74 ± 0.00	20.23 ± 0.00
		N1 (30 SDU)	11.15 ± 0.00	32.07 ± 0.00
Dexpanthenol	Corneregel^®^ (Bausch+Lomb Incorporated, Berlin, Germany)	N1 (10 SDU)	11.81 ± 0.00	13.00 ± 0.01
		N1 (30 SDU)	13.36 ± 0.00	37.01 ± 0.01
		N2 60 (SDU)	18.07 ± 0.00	69.45 ± 0.03
		N3 (120 SDU)	26.43 ± 0.00	138.86 ± 0.06
Hypromellose	Artelac^®^ (Bausch+Lomb Incorporated, Berlin, Germany)	N1 (10 SDU)	11.48 ± 0.00	34.44 ± 0.00
		N1 (30 SDU)	34.44 ± 0.00	38.34 ± 0.00
		N2 (60 SDU)	18.14 ± 0.00	76.67 ± 0.00
		N3 (120 SDU)	36.28 ± 0.00	153.34 ± 0.00
**B**				
Hyaluron	Vismed® (TRB Chemedica, Staffordshire, UK	N1 (10 ml) (#)	2.83 ± 0.00	6.04 ± 0.00
		N1 (15 ml)	6.87 ± 0.00	7.67 ± 0.00
		N3 (3 × 10 ml) (#)	19.44 ± 0.00	17.91 ± 0.00
		N3 (3 × 15ml)	10.59 ± 0.00	23.54 ± 0.00
	HYLO^®^ (URSAPHARM Arzneimittel GmbH, Saarbrücken, Germany)	N1 (10 ml) (#)	10.57 ± 0.00	17.66 ± 0.00
		N3 (2 × 10 ml) (#)	13.07 ± 0.00	38.27 ± 0.01
Euphrasia	Euphrasia^®^ (WELEDA AG, Arlesheim, Switzerland)	N1 (10 ml) (#)	7.19 ± 0.00	9.64 ± 0.00
Dexpanthenol	Corneregel^®^ (Bausch+Lomb Incorporated, Berlin, Germany)	N1 (10 ml)	4.75 ± 0	6.38 ± 0
		N3 (3 × 10 ml)	8.60 ± 0	19.28 ± 0
Hypromellose	Artelac^®^ (Bausch+Lomb Incorporated, Berlin, Germany)	N1 (10 ml)	4.81 ± 0	6.36 ± 0
		N3 (3 × 10 ml)	8.72 ± 0	19.59 ± 0.01

### Waste analyses per product

All products for the treatment of DED listed in the PharMaAnalysis database were obtained, and the packaging, the instruction leaflet, and the container (e.g., dropper bottle, MDU, SDU) were separated. Products containing ciclosporin A (Ikervis^®^), some hyaluronic acid products (Vismed^®^), and hydrocortisone (Softacort^®^) contain an additional inner packaging. This inner packaging consists of a metal (probably aluminum)-plastic compound material, for light protection. We suppose that the CO_2_ equivalent generated during the production and recycling of this compound material is likely to be at least as high, but probably even significantly higher, than for plastic consisting of one component [18–20]. Thus, we have analyzed this material together with the other plastics. Each drop container was emptied and dried with compressed air. Every component (except for the container content) was measured three times using a precision scale (KERN ABJ 220-4NM), and the average was calculated (Table 1).

With the list of prescription numbers (sorted by year, name, packaging size, prescription number, and costs), the total paper and plastic waste weight was calculated for the years 2016 to 2021.

### Calculation of 30-day therapy waste and statistics

We assumed that one pack of 30 SDU and one MDU are each used for 1 month. With the paper and plastic weight of the different prescription sizes, the average waste for a 30-day therapy was calculated as follows: For the SDU waste, the paper and plastic weight per SDU was calculated and then multiplicated with 30 days.

Formula for SDU:

$$\frac{\text{Paper}/\mathrm{plastic}\;\mathrm{weight}\;\mathrm{per}\;\mathrm{package}\;\lbrack\text{g}\rbrack}{\mathrm{prescription}\;\mathrm{size}\;\lbrack\text{N}1-3\rbrack}\mathrm x30=\mathrm{paper}/\mathrm{plastic}\;\mathrm{waste}\;\mathrm{per}\;\mathrm{month}$$

e.g., 30-day waste for paper weight for Vismed^®^:

$$\frac{45\mathrm g}{120}\mathrm x30=34.2\;\mathrm g$$

A formula for MDU was not necessary. The measured weight data were directly used for the 30-day waste assumption (1 MDU ≙ 1 month).

Prism 9.0 (GraphPad, La Jolla, CA, USA) was used for statistical analysis and graphs. For comparison of SDU vs. MDU (without filter) and MDU (without filter) vs. MDU (with filter), the Mann-Whitney tests were performed. Cochrane-Armitage test for trend was used to evaluate a possible increase in the incidence. We considered a *p*-value ≤ 0.05 to be statistically significant.

## Results

### Prescription data

**Fig. 1:**
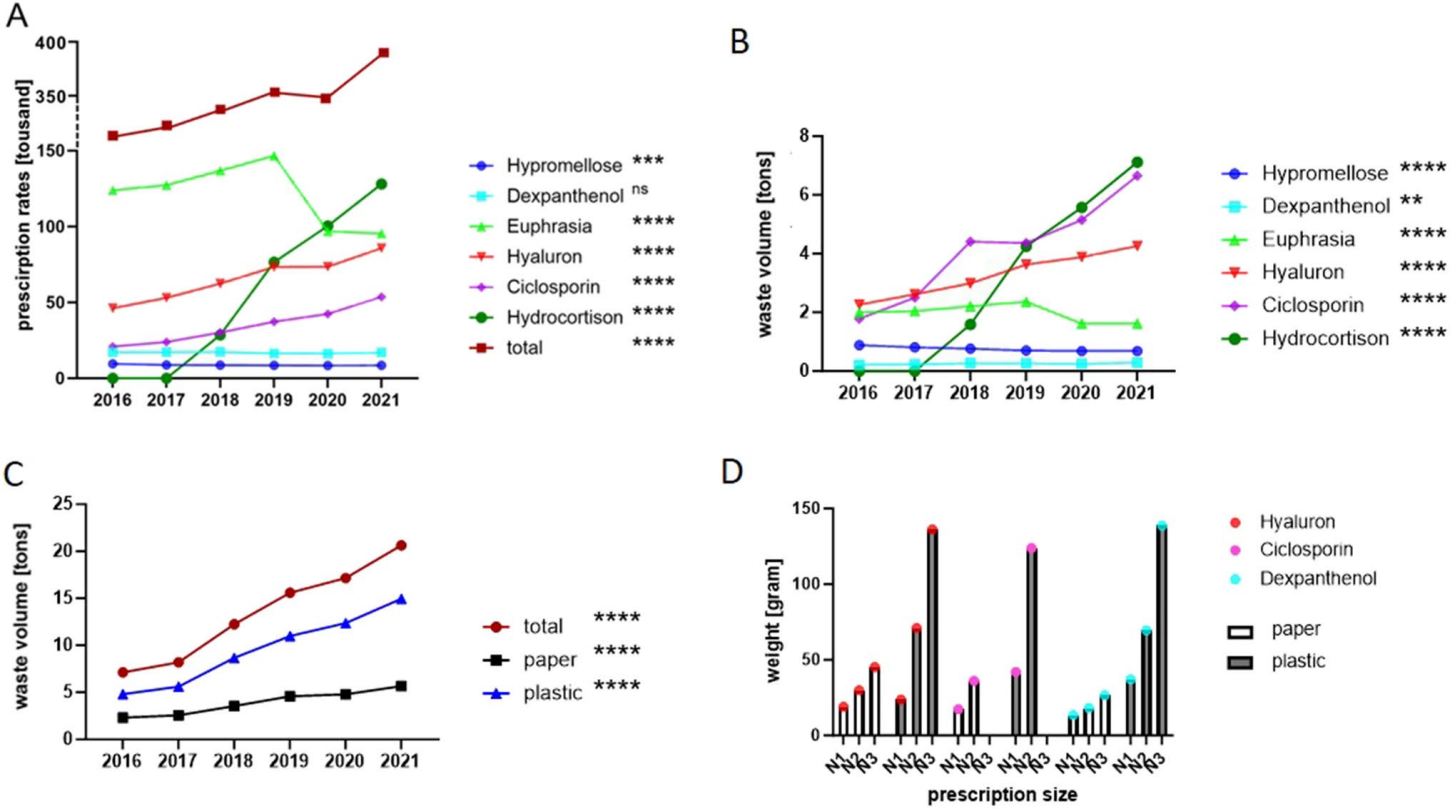
**A** Development of prescription numbers in 2016 to 2021 for each ingredient and the total number. **B** Combined waste volume of paper and plastic in the years 2016 to 2021 for six different DED product ingredients (seven different products). **C** Waste volume for paper and plastic per year for all products together. *P* values were created with the Cochrane-Armitage test for trend to evaluate a possible increase in the incidence. ****p < 0.0001, ***p = 0.0004, **p = 0.0018; ns = non-significant. **D** Development of paper and plastic waste for different prescription/packaging sizes, exemplary for hyaluronic acid, ciclosporin, and dexpanthenol. For hydrocortisone, only one packaging size was prescribed. We compared the paper volume with the plastic volume of different packaging sizes (N1–N3). N1 ≙ the smallest, N2 ≙ the middle and N3 ≙ the biggest prescription/packaging size of a product. This figure shows that the paper volume per package does not rise to the same extent as the plastic volume per package for a bigger size. The larger the package size, the less paper waste per unit

The total number of prescribed product units (in thousand) increased significantly from 2016 to 2021 from 217.90 to 388.60 (*p* < 0,0001, Fig. 1A), equivalent to an increase of 78%. While prescriptions of hypromellose decreased significantly (2016: 9.60 to 8.60 in 2021; *p* = 0.0004) dexpanthenol remained stable (2016: 17.30 to 2021: 17.00, *p* = 0.0835, ns). Euphrasia increased significantly until 2019 (2016: 123.90 to 2019: 146.70) and then showed a rapid reduction (2021: 95.40; *p* < 0.0001). Numbers of prescription for hyaluronic acid and ciclosporin A showed a constant increase (hyaluronic acid in 2016: 46.20 to 2021: 85.80; *p* < 0.0001; ciclosporin A 2016: 20.90 to 2021: 53.70; *p* < 0.0001). Hydrocortisone prescriptions first began in 2018 and steadily rose from 28.60 to 2021 128.10 thousand packages (347.9%, *p* < 0.0001) (Fig. 1A).

### Waste data

In the years 2016 to 2021, the annual total waste volume (paper and plastic) of prescription-based tear substitute use increased (*p* < 0.0001) from 7.13 t (2.32 t paper and 4.82 t plastic) in 2016 to 20.64 t (5.68 t paper and 14.95 t plastic) in 2021 (Fig. 1B), which is equivalent to increase of 189%. Paper waste increased from 2.32 t in 2016 to 5.68 in 2021 by 144.8 % (*p* < 0.0001), while plastic waste increased from 4.82 t in 2016 to 14.95 t in 2021 or 210.2% (*p* < 0.0001).

#### Increasing waste volume

Over the years 2016 to 2021, for dexpanthenol, an increase in paper by 25.8% and in plastic by 41.3% was found (paper: 2016: 0.06 t vs. 2021 0.08 t; *p* = 0,199; plastic: 2016: 0.15 t plastic vs. 2021: 0.21 t; *p* = 0.0031, total waste volume 2016 vs. 2021 *p* = 0.0018) (Figure 1B). Hyaluronic acid products showed a higher waste rate of 53.6% for paper and 52.9% for higher plastic volume in 2021 in comparison to 2016 (paper 2016 0.634 t vs. 2021 1.19 t; *p* < 0.0001; plastic 2016 1.63 t vs. 2021 3.08 t; *p* < 0.0001) (Figure 1B). For ciclosporin A, a rise of waste volume for paper of 340.1% and for plastic of 385.3% could be detected (paper: 2016 0.46 t vs. 2021 1.58 t; *p* < 0.0001; plastic: 2016 1.32 t; 2021 5.08 t; *p* < 0.0001) (Figure 1B). The biggest increase was seen for hydrocortisone (paper: 2016 0 t vs. 2021 1.93 t; increase of 448.4 %; *p* < 0.0001; plastic: 2016 0 t vs. 2021 5.19 t, increase of 448.0%, *p* < 0.0001) (Figure 1B).

#### Decreasing waste volume

Hypromellose showed a significant decrease in waste volume of 16.6% for paper and 24.2% in plastic waste (paper 2016: 0.20 t vs. 2021 0.17 t; *p* = 0.0320; plastic 0.69 t vs. 2021: 0.52 t; *p* < 0.0001) (Figure 1B). For tear substitute products based on euphrasia, a reduction of waste volume over the 6 years was shown, too: paper 2016 0.96 t vs. 2021 0.80 t (decrease of 16.67 %, *p* < 0.0001) and plastic 2016 1.04 t vs. 2021 0.88 t plastic (decrease of 15.4%, *p* < 0.0001) (Figure 1B).

#### Waste for a 30-day therapy

The average amount of paper waste resulting from a 30-day treatment for one patient was calculated to be 24.3 ± 18.7 g for a SDU and 4.8 ± 1.7 g for MDU without filter (*p* = 0.0003). The mean plastic weight was 35.0 ± 4.0 for SDU and 6.6 ± 0.7 g for a MDU without filter (*p* < 0.0001). A thirty-day use of MDUs with a filter system resulted in a higher waste volume of paper (8.8 g ± 1.7 g) and plastic (15.1 ± 5.8 g), which were both higher than for unfiltered products (paper *p* = 0.0190, plastic *p* = 0,1143), but both significantly lower than for SDUs (paper *p* = 0.0034, plastic *p* < 0.0001).

## Discussion

There is no information/literature regarding recycling rates of eyedrop containers until now. However, the recycling rate of plastic waste in 2021 was described by the Nature Conservation Union Germany (NABU, Conversio Study, Society for Packaging Market Research, Federal Environment Agency, 2022) with only 60%, i.e., 3.2 million tons of plastic. The recycling rate for paper (packagings) in Germany is specified by the Federal Environment Agency in 2020 with 74,3%, i.e., 13.97 million tons of packaging waste.

In this analysis, we were able to show that the prescription-based therapy of DED (including lubricants) causes a high total waste weight, which also increased substantially between 2016 and 2021. Especially plastic waste volume rose by 189% in these 5 years. Preparations with the ingredients hyaluronic acid, ciclosporin A, and hydrocortisone contributed the largest amounts of total plastic waste. This effect predominantly resulted from rising prescription figures for products containing hydrocortisone, hyaluronic acid, and ciclosporin A. The additional material of the inner packaging of Ikervis^®^, Vismed^®^, and Softacort^®^ may have also contributed to this effect.

It is likely that the decrease in prescription rates and waste volume of euphrasia, dexpanthenol, and hypromellose in 2019 and 2020 were the results of the market launch of a new low-dose hydrocortisone product on 27th October 2017. Euphrasia was marketed as an anti-inflammatory eye drop, which soothes the eye when there is irritation as well as redness and allows inflammation to subside (package insert). Hydrocortisone is a potent and effective agent and may be prescribed in low doses as an alternative to euphrasia, dexpanthenol, and hypromellose [21–23].

Another interesting finding is the significant and constant increase (*p* < 0.0001) of the total prescriptions and thus the waste volume of ciclosporin A. Albeit ciclosporin A has long been known to be a safe and effective anti-inflammatory treatment for DED [24, 25], it first gained official market access as a commercially available product for the topic use in DED in Germany on 19th March 2015. The number of prescriptions documents the increasing use of this product. Although ciclosporin A only reached approximately half of the absolute prescription numbers for hydrocortisone, the waste volume for paper and plastic of those two products was not significantly different in the years 2019, 2020, and 2021 (Figure 1B). This is caused by the larger package size of ciclosporin A prescriptions, as the detailed annual PharMaAnalyst data shows (e.g., in 2021 hydrocortisone 128,100 packages with 30 SDU ≙ N1, ciclosporin A 19,200 packages with 30 SDU ≙ N1, and 34,500 packages with 90 SDU ≙ N2; the final amount of SDUs prescribed equals around 3.8 and 3.7 million for hydrocortisone and ciclosporin A, respectively).

As shown in Figure 1D, the paper weight per package does not rise to the same extent as the plastic weight per package for a bigger prescription size. This effect and the annually differing prescription sizes (e.g., increasingly for ciclosporin A N3) explain the different developments in the increase of paper waste compared to plastic waste over the years.

A limitation of our analysis might be, that—in order to calculate an average waste production during 30 days of therapy—we assume that 30 SDU and one MDU are used for 1 month. Possibly, some patients extend the use of SDU/MDU or even use several SDU per day or MDU per month, respectively. Therefore, in a follow-up project in our Dry Eye outpatient clinic, we will evaluate the real period of use and waste volume per 30-day therapy.

Our data show a rising number of prescriptions and waste weight volume. It could be argued that the COVID-19 pandemic might have contributed to the rising prescription numbers. Since currently in PharMaAnalyst there is no post-pandemic data available (currently last year in the database: 2021), we are not able to evaluate whether the COVID-19 pandemic influenced the prescription data. Furthermore, as our data shows steadily increasing prescription rates over 3 years prior to the pandemic, we consider this effect to be negligible.

Another important limitation of our analysis is that only products that are prescribed to patients with statutory health insurance are listed in the PharMaAnalyst database. Over-the-counter products (OTC) that do not require a prescription are not considered. As very few dry eye patients receive drops on prescription we assume, that the real total waste generated by OTC dry eye therapy is probably several times higher. Furthermore, also the analysis of products with a mandatory prescription (hydrocortisone, ciclosporin A) only covers 83 % of the German population. For this reason, we estimate that the total waste generated by products with a mandatory prescription is around 20% higher. Unfortunately, there is no data available about patients in the other insurance groups (private insurance, social welfare recipients, uninsured). In order to gain more information about possible differences in those groups and to collect real-world data about the use, cost, and insurance coverage of DED associated therapy, as well as the waste generated by those products, we already began a prospective cross-sectional study in our subspeciality dry eye clinic.

One further limitation is that the analysis of the number of prescribed packages or daily doses could lead to an overestimate of waste volume due to unfilled prescriptions. Unfortunately, there is no data about the number of unfilled prescriptions via PharMaAnalyst, and to our knowledge, there is currently no way to track the filling of prescriptions in Germany.

As our data clearly shows choosing an MDU can be a simple first step towards limiting the use of resources and the production of waste with DED treatment. Products in MDUs with filter systems have a significantly higher weight than those in MDUs without filter system. Interestingly in the cohort of products analyzed by us, this was observed not only for the plastic weight, but also the paper waste weight. Here, companies have a choice and may easily be able to minimize the use of paper.

QR-coded links to product information would further reduce the use of paper and lead to a more sustainable and environmentally conscious packaging option. Furthermore, to decrease the waste of paper in clinical practice, prescriptions and product information could be transmitted to the electronic medical record and be available to the patients on every PC, smartphone, or connected device. But a more thorough analysis of required resources (e.g. server and electricity) is required first. A balanced approach to dropper bottle choice is also warranted. Products in filterless MDUs require the addition of preservatives, which have other potential detrimental effects on the environment [26]. Using a MDU with an integrated filter may thus be the best compromise in terms of an ecologically sustainable packaging. This analysis of the current state of waste production for the first time provides specific figures on this so far ignored aspect of DED therapy. An ecologically sustainable, climate-neutral ophthalmology is the goal for the coming decades [27]. While the attempt at waste reduction may seem justified, the safety and efficacy of the treatment must remain the primary goal of patient care. To our knowledge, so far, no medical concern of using MDUs with filters compared to SDUs has been reported. A study which compared a single-dose and multi-dose system stated that the convenience of opening and applying eye drops and the number of drops retrieved were substantially better for multi-dose systems [28]. There is evidence that the use of MDUs with filters reduces contamination rates related to insufficient preservatives in MDUs without filters and optimized nozzle geometry. This suggests that MDUs with a filtration system may be superior to MDUs with preservatives but no filter [29, 30].

We hope that our findings can contribute a first step to a sustained reduction in CO_2_ emissions and reduce negative ecological effects potentially associated with the field of dry eye therapy. Further evidence and research are needed to generate additional knowledge not only on other forms of DED treatment, such as OTC products or single-use devices [31], but also on the impact of high-end diagnostic tools such as tear film interferometers and placido-based devices [32, 33].
